# Diagnostic Accuracy of Lung Ultrasound for Pneumonia in Acutely and Critically Ill Neonates, Children, and Young Adults: A Systematic Review and Meta-Analysis

**DOI:** 10.3390/diagnostics15243122

**Published:** 2025-12-08

**Authors:** Carmina Guitart, Judit Becerra, Sara Bobillo-Perez, Josep L. Carrasco, Gonzalo Peon, Monica Balaguer, Iolanda Jordan

**Affiliations:** 1 Paediatric Intensive Care Unit, Hospital Sant Joan de Déu, University of Barcelona, Passeig de Sant Joan de Déu, 2, Esplugues de Llobregat, 08950 Barcelona, Spain; carmina.guitart@sjd.es (C.G.); judit.becerra@sjd.es (J.B.); sara.bobillo@sjd.es (S.B.-P.); yolanda.jordan@sjd.es (I.J.); 2 Immunological and Respiratory Disorders in the Paediatric Critical Patient Research Group, Institut de Recerca Sant Joan de Déu, University of Barcelona, 08007 Barcelona, Spain; 3 Biostatistics Unit, Department of Basic Clinical Practice, University of Barcelona, 08007 Barcelona, Spain; jlcarrasco@ub.edu (J.L.C.);; 4 Faculty of Medicine, University of Vic-Central University of Catalonia (UVic-UCC), 08500 Vic, Spain; 5 Medical and Surgical Department, Medicine School, Campus Clinic, University of Barcelona, 08007 Barcelona, Spain; 6 Paediatric Infectious Diseases Research Group, Institut de Recerca Sant Joan de Déu, Consorcio de Investigación Biomédica en Red de Epidemiología y Salud Pública (CIBERESP), 08950 Barcelona, Spain

**Keywords:** pneumonia, lung ultrasound, paediatrics, critical care

## Abstract

**Background:** Pneumonia remains a major cause of morbidity and mortality among critically ill children. Lung ultrasound has emerged as a promising bedside diagnostic tool. **Methods**: A systematic review and meta-analysis across PubMed, Embase, The Cochrane Library, Scopus, World Health Organization Libraries, Epistemonikos, and MedRxiv was conducted to evaluate the diagnostic accuracy of lung ultrasound for pneumonia in paediatric patients. Publication bias was evaluated using the generalised Egger’s test. Diagnostic performance metrics, including sensitivity, specificity, and the area under the receiver operating characteristic curve were pooled using a bivariate random-effects model. **Results:** Thirty studies comprising a total of 4356 children were included. The studies were of high methodological quality, with minimal heterogeneity. Lung ultrasound pooled sensitivity was 91% (95% CI: 87–94%), and specificity was 90% (95% CI: 83–94%). The ROC curve was 0.95 (95% CI: 0.90–0.95), indicating excellent diagnostic performance. **Conclusions:** LUS is a reliable and accurate imaging modality for diagnosing pneumonia in critically ill children. The findings support its use as a first-line diagnostic tool in emergency and intensive care settings. PROSPERO Research registration number: CRD42021223679.

## 1. Introduction

Pneumonia is the leading infectious cause of morbidity and mortality in children worldwide [1,2], responsible for over 740,000 deaths annually in those under five years of age [3]. Despite its high incidence and potential for serious complications, timely and accurate diagnosis remains challenging, often requiring multiple complementary tests. This highlights the pressing need for rapid, reliable, and accessible diagnostic methods.

In paediatric populations, pneumonia is a common cause of admission to intensive care units (PICU) [4,5], particularly in high-income countries, where it contributes significantly to healthcare costs and widespread antibiotic use [6]. However, no universally accepted diagnostic reference standard currently exists. Clinical presentations vary with age and causative pathogen, further complicating its diagnosis [7,8,9].

Chest X-ray (CXR) is commonly used in clinical practice, but has well-documented limitations in detecting pneumonia, distinguishing between viral and bacterial aetiologies, and predicting clinical outcomes [10,11,12,13,14]. Computed tomography (CT), while more accurate, is not routinely used due to concerns about radiation over exposure [15], need for patient cooperation or sedation, and higher cost constraints—issues particularly relevant in resource-limited settings [1,16].

Lung ultrasound (LUS) has emerged as a promising alternative: it is safe, portable, non-invasive, cost-effective, and increasingly used even in primary care for the evaluation of multiple pulmonary conditions. It offers real-time bedside imaging and avoids many of the drawbacks associated with CXR and CT. However, uncertainty remains regarding its diagnostic accuracy, particularly in critically ill paediatric patients [17,18].

Given these gaps, the present systematic review and meta-analysis aims to comprehensively synthesise the current evidence on the diagnostic accuracy of LUS for pneumonia in this high-risk population. The objective is to assess its clinical utility in emergency and intensive care settings, where timely and precise diagnosis is essential.

## 2. Material and Methods

### 2.1. Study Design and Protocol Registration

This systematic review and meta-analysis followed the PRISMA (Preferred Reporting Items for Systematic Reviews and Meta-analyses) guidelines. The study protocol was registered in the PROSPERO database (Registration Number: CRD42021223679, date created: 30 November 2020).

### 2.2. Data Sources and Search Strategy

A comprehensive literature search was conducted across several databases, including PubMed, Embase, The Cochrane Library, Scopus, World Health Organization Libraries, Epistemonikos, and MedRxiv, covering all studies published until December 2022.

The search strategy was developed in collaboration with a hospital expert librarian to maximise sensitivity and relevance. Search terms included combination of keywords such as paediatrics (“age < 21 years”), clinically suspected pneumonia (“pneumonia”), and imaging tests for diagnosing clinically suspected pneumonia (“ultrasound,” “sonography,” “ultrasonography,” “radiography,” “chest film,” “chest radiograph,” “computerized tomography,” or “image test”). Reference lists of included studies, related reviews, and articles suggested by PubMed and EMBASE were manually screened to identify additional relevant publications.

### 2.3. Eligibility Criteria

Studies were selected according to predefined eligibility criteria based on PICO (Population, Interventions, Comparator, and Outcome). Eligible studies included paediatric patients from 0 to 21 years of age with clinical suspicion or confirmed diagnosis of pneumonia, evaluated in emergency departments (acutely ill patients) or critical care settings (critically ill patients requiring intensive care support). Studies focusing only on neonatal or adult populations were excluded to maintain homogeneity within the paediatric cohort.

The intervention of interest was LUS performed for pneumonia diagnosis. Eligible studies were required to compare LUS findings against a reference standard, which could include clinical evolution, CXR, CT or a composite diagnostic criterion based on clinical presentation, imaging, laboratory markers, and/or microbiological findings [19].

Studies were included only if they reported sufficient data to reconstruct 2 × 2 contingency tables, enabling the calculation of diagnostic performance metrics.

The methodological quality and risk of bias of the diagnostic accuracy studies included in this review were assessed using the QUADAS-2 tool [20] (Whiting et al., 2011). QUADAS-2 evaluates four key domains: Patient Selection, Index Test, Reference Standard, and Flow and Timing. Each domain was assessed for risk of bias (classified as low, high, or unclear). The assessment was based on signalling questions provided by the QUADAS-2 framework, which guides reviewers in making judgments about potential sources of bias and applicability concerns. The results are summarised in frequency tables and visualised using bar plots to illustrate the distribution of risk levels across domains. Only original, peer-reviewed studies published in English or Spanish were considered.

Studies were excluded if they lacked a clear diagnostic standard, used inappropriate or poorly described methodologies (e.g., absence of randomization or blinding where relevant), or failed to report outcomes in a manner compatible with meta-analytic synthesis. Review articles, meta-analyses, editorials, letters, case reports, and conference abstracts were also excluded.

Studies were classified as high risk for bias if they lacked a detailed description of randomization. Incorrect randomization methods, such as coin tosses, were exclusionary. Only articles in English and Spanish were considered.

The primary outcome was the diagnostic accuracy of LUS in identifying pneumonia. Accuracy was assessed through sensitivity, specificity, predictive values, and likelihood ratios.

### 2.4. Study Selection

**Figure 1:**
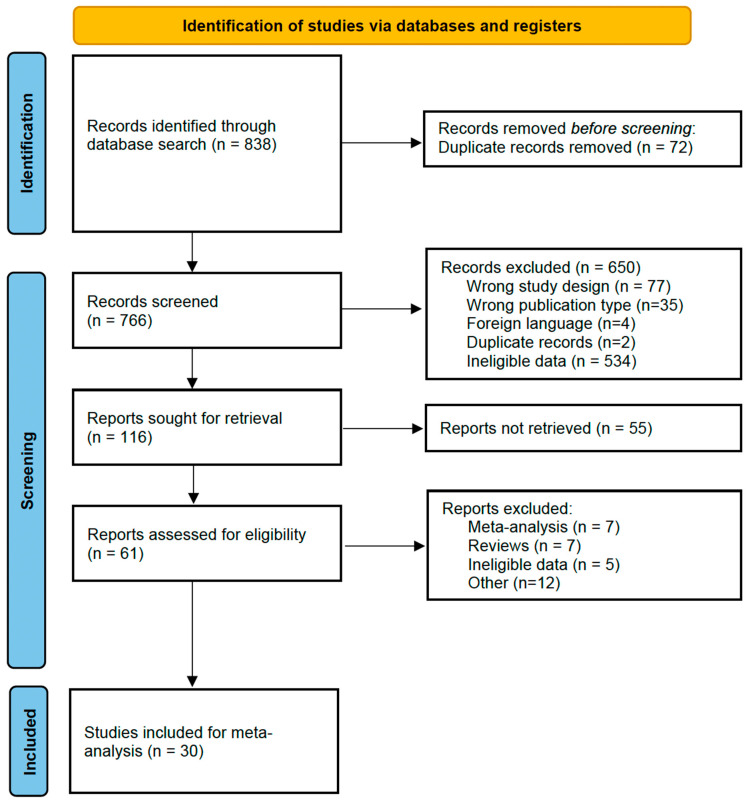
Flow diagram for study selection.

All identified records were imported into Rayyan QCRI [21], and duplicates were removed. Titles and abstracts were screened for relevance, followed by full-text review. Each article was evaluated independently by four reviewers (CGP, SBP, MBG, IJG), with disagreements resolved through discussion and consensus. A PRISMA flow diagram illustrates the selection process (Figure 1).

### 2.5. Data Extraction and Management

Data were extracted independently by four reviewers (CGP, SBP, MBG, and IJG) using a standardised form that included the following items:

1. Study characteristics: Author, year, country, setting, sample size, design.
2. Study design: Randomised, blinded, prospective/retrospective.
3. Participant characteristics: Age range, sex, clinical setting.
4. Diagnostic method details: LUS equipment and protocols, probe type, scanning zones, operator expertise, blinding procedures, follow-up.
5. Outcome data: True positives, false positives, false negatives, true negatives, and statistical measures of diagnostic accuracy.
6. Adverse events and other relevant findings.

Where necessary, corresponding authors were contacted to obtain missing data.

### 2.6. Risk of Publication Bias Assessment

Publication bias was evaluated using the generalised Egger’s test for diagnostic accuracy studies, as proposed by Hong (2020) [22], applied jointly to sensitivity and specificity estimates. The test was implemented through a parametric bootstrap approach to improve robustness. In addition, visual inspection of funnel plots for sensitivity and specificity was performed to assess asymmetry.

### 2.7. Statistical Analysis

Statistical analyses were conducted using the meta v8.2.1 [23], metafor 4.8.0 (2010) [24], and mada 0.5.12, R v4.5.0 packages. Diagnostic accuracy was assessed using sensitivities, specificities, likelihood ratios, and diagnostic odds ratio (DOR). A random-effects model was applied to calculate pooled effect estimates and corresponding 95% confidence intervals using the DerSimonian–Laird method [25]. Between-study heterogeneity was evaluated using Cochran’s Q statistic [26], Higgins’ I^2^ [27], and prediction intervals for pooled effects [28]. A bivariate model approach [29] was employed to jointly model sensitivity and specificity. Sensitivity and specificity estimates and their confidence intervals (continuity-corrected estimates and Wald intervals) were computed using the madad function of the R library mada [30], while the pooled estimates are the previously presented estimates from the bivariate model (Reitsma function of the same R library).

The summary estimates for both log-Diagnostic odds ratios and the likelihood ratios are those from the bivariate model presented previously generated through the sampling procedure proposed in Zwinderman and Bossuyt [31]. The individual study estimates and confidence intervals were obtained in the same manner as for the sensitivity and specificity. The weights displayed are derived from a univariate random effect size estimation of the diagnostic odds ratio, as in DerSimonian and Laird [25].

Differences in diagnostic performance were thoroughly examined using Summary Receiver Operating Characteristic (SROC) curves. The differences in diagnostic performance were tested using bootstrap technique applying the summary AUC comparison test for Summary ROC curves, as proposed by Noma, Matsushima, and Ishii [32], and implemented in the R library *dmetatools* [32]. The Summary ROC curve approach developed by Rutter and Gatsonis [33] was used to compare the AUCs of the bivariate models.

Forest plots of the sensitivity and specificity, log diagnostic odds ratios, and likelihood ratios for both LUS and comparators are provided for the articles included on the bivariate analysis. The forest plots were generated using the R library *forestploter* [34].

**Dealing with missing data**: If there were missing data, we attempted to contact the corresponding authors of included studies for any necessary data by e-mail. However, if the missing data could not be obtained, the study was excluded from the analysis.

**Subgroup analysis**: If there was significant heterogeneity between studies, a subgroup analysis was carried out to explore the causes of heterogeneity.

## 3. Results

### 3.1. Study Selection

The initial database search yielded 838 records (Figure 1). After removal of 72 duplicates, 766 titles and abstracts were screened, leaving 116 articles for full-text review. Following the exclusion of 55 articles (14 reviews/meta-analyses, 5 adult-only studies, 12 for methodological or reporting reasons, and others), 30 studies met the inclusion criteria and were evaluated using the QUADAS-2 tool. The main source of potential bias was patient selection. Articles were assessed by at least two independent reviewers. A third evaluator reassessed the articles when there were discrepancies. Four case-only studies were excluded from the pooled bivariate analysis due to incompatibility with the model.

### 3.2. Characteristics of Included Studies

The 30 eligible studies, published between 2008 and 2022, involved a total of 4356 paediatric patients. The median sample size was 100 (IQR 69–154), with participants ranging in age from 0 to 21 years. Most studies (63%) were prospective cohorts, and 60% were performed in emergency department settings. Geographically, the studies were conducted in Italy (*n* = 8), the United States (*n* = 4), Spain (*n* = 1), China (*n* = 1), and other countries.

Regarding comparators, CXR was the most frequent reference standard in 22 (73%) studies. Regarding LUS features, pulmonary consolidation was the most assessed finding in 29 (96.6%) studies. Regarding quality measures, 24 studies (80%) reported blinding of operators to comparator outcomes.

Full details of demographics, imaging protocols, and diagnostic outcomes are presented in Table 1, Table 2 and Table 3.

### 3.3. Pooled Diagnostic Accuracy

This meta-analysis demonstrated that LUS had excellent diagnostic performance for LUS (see Table 4).

**Figure 2:**
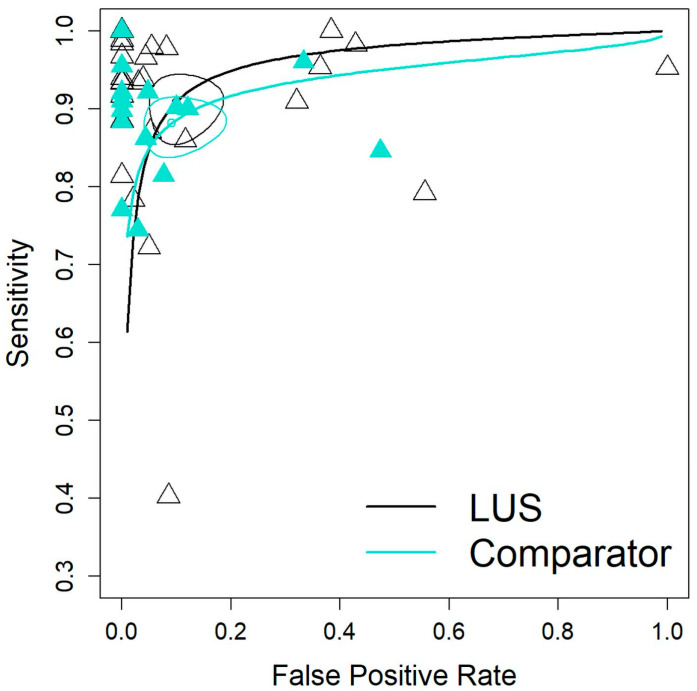
Comparison of diagnostic performance between LUS and the comparator test using summary ROC curves. Each triangle represents the accuracy of each article. Ellipses represent confidence regions around the average sensitivity and specificity estimates for each test. The overlap between ellipses suggests that the diagnostic accuracy of the two tests may be similar.

The pooled sensitivity (Se) and specificity (Sp) for LUS were 0.91 (95% CI: 0.87–0.94) and 0.90 (95% CI: 0.83–0.94), respectively. The area under the curve (AUC) for LUS resulted of 0.95 (95% CI: 0.90–0.95) indicating strong overall performance (Figure 2).

Comparators showed an Se of 0.88 (95% CI: 0.85–0.91) and Sp of 0.91 (95% CI: 0.83–0.95). Heterogeneity was low for both groups (LUS: I^2^ = 8.5%; CXR: I^2^ = 3.5%), indicating a high degree of homogeneity among the included studies.

### 3.4. Forest Plot Analysis

**Figure 3:**
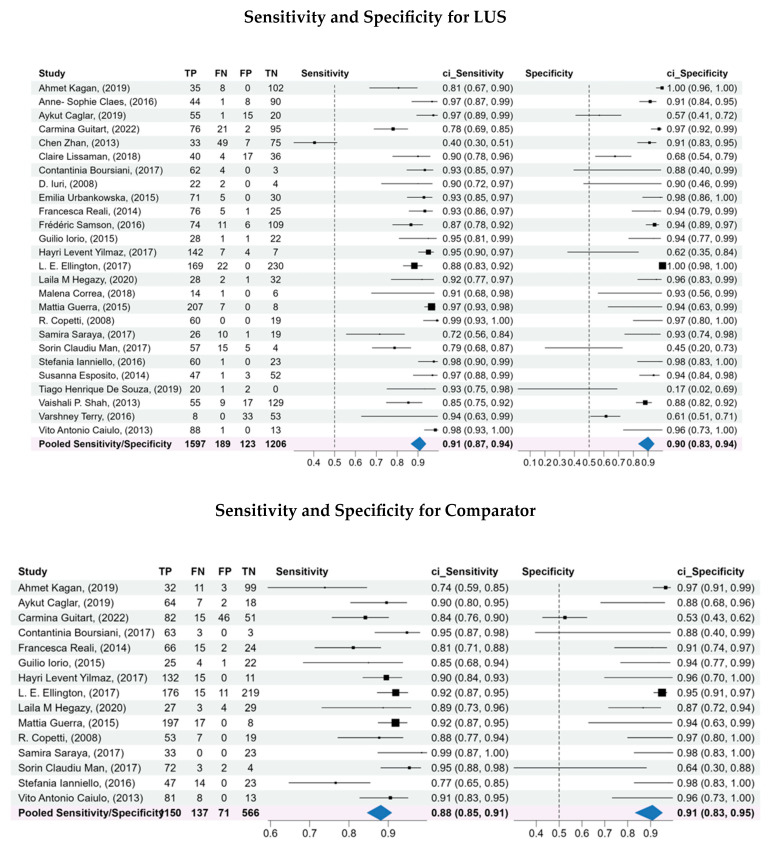

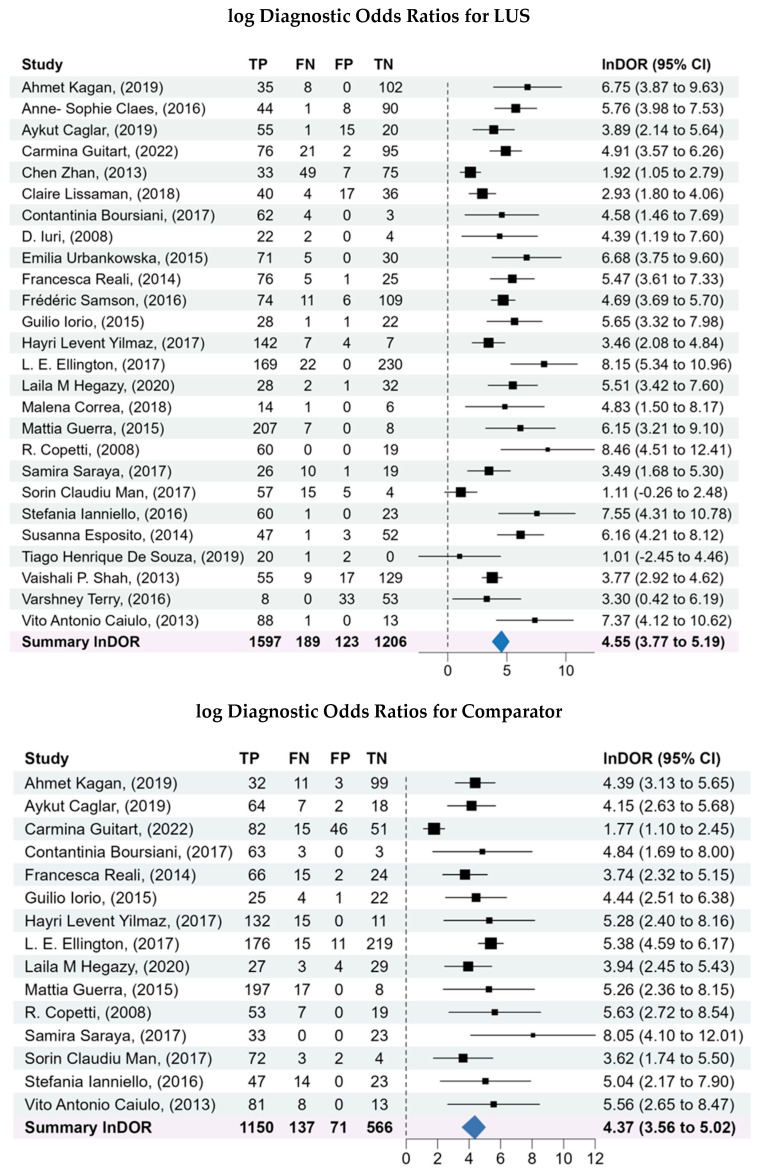

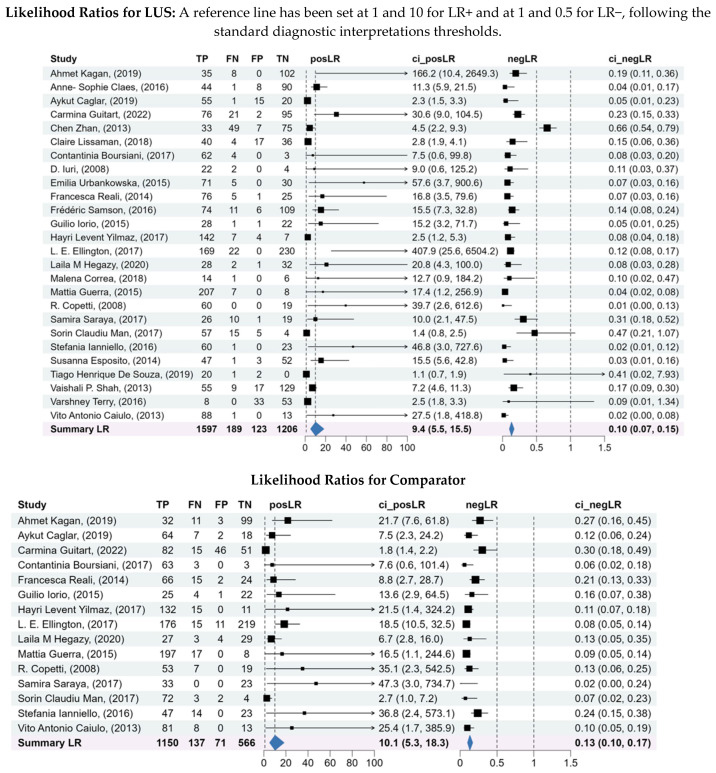
Forest plots of the sensitivity and specificity, log diagnostic odds ratios, and likelihood ratios for both LUS and comparator. A bivariate analysis was conducted to assess the overall diagnostic capability of LUS compared to the comparators for diagnosis of pneumonia. A bivariate model was used to pool the sensitivity, specificity, log diagnostic odds ratios, and likelihood ratios of the included studies, considering the inherent correlation between these two measures of diagnostic accuracy.

Forest plots (Figure 3) confirm the consistent sensitivity for LUS across studies, with relatively homogenous estimates. Specificity values display more variability, but remain consistently high overall. In comparison, CXR shows more variable sensitivity across studies, whereas specificity estimates are more stable.

### 3.5. Diagnostic Odds Ratios (DOR)

The pooled lnDOR for LUS was 4.55 (95% CI: 3.77–5.19), reflecting a strong association between positive LUS findings and pneumonia. The comparator lnDOR was slightly lower at 4.37 (95% CI: 3.56–5.02).

### 3.6. Likelihood Ratios

Pooled likelihood ratios further supported the diagnostic value of LUS. Positive LR resulted in 9.44 (95% CI: 5.48–15.5), demonstrating strong rule-in capability. Negative LR resulted in 0.10 (95% CI: 0.07–0.15), indicating reliable rule-out performance.

In contrast, comparators LR estimates were more heterogeneous, particularly in LR–values, suggesting that CXR was less consistent in excluding pneumonia compared to LUS. Details are shown in Figure 3.

## 4. Discussion

This meta-analysis confirms that LUS is a reliable, accurate, and clinically useful diagnostic tool for pneumonia in critically ill children. Our findings align with prior meta-analyses conducted in both paediatric and adult populations, which consistently reported high sensitivity and specificity for LUS compared with conventional imaging modalities [1,16,19,64,65,66,67]. Importantly, by focusing exclusively on paediatric patients in emergency and intensive care settings, our study reduces heterogeneity (I^2^ = 8.5%) and offers more targeted evidence relevant to high-acuity clinical scenarios compared to earlier studies. It also includes a larger population than most previous meta-analyses.

Across 30 studies and over 4300 patients, LUS achieved a pooled sensitivity of 91% and specificity of 90%, with excellent overall performance (AUC = 0.95). In other individual studies [1,67], LUS consistently demonstrated superior Se compared to CXR, highlighting LUS’ high diagnostic precision. These values confirm LUS as a powerful tool for both ruling in and out pneumonia, with strong positive [39,53] and negative likelihood ratios [17,41]. Compared to CXR, which showed slightly lower sensitivity but comparable specificity, LUS demonstrated superior consistency and reliability across settings [19,66]. Yan et al. further emphasised CXR limitations, noting its lower Se (91%), suggesting that CXR may miss mild or early-stage pneumonia cases [66].

Several individual studies highlight the particular strengths of LUS. Caiulo et al. reported sensitivity approaching 99%, emphasising its ability to detect small consolidations that may be missed on CXR [39]. Iuri et al. and others further demonstrated LUS’s advantage in detecting pleural effusions and pneumonia-related complications, underscoring its role as a comprehensive bedside imaging modality [37]. Moreover, the repeatability and radiation-free nature of LUS make it ideal for monitoring disease progression and treatment response, something CXR and CT cannot provide without exposing patients to additional risks.

These findings solidify LUS as a clinically valuable tool, particularly in resource-limited settings where rapid, reliable diagnostics are crucial. By ensuring accurate case identification, LUS aids in optimising treatment decisions, potentially reducing unnecessary antibiotic use and improving patient outcomes. Its high diagnostic accuracy minimises uncertainty, a key challenge in emergency and intensive care settings.

The meta-analysis results are broadly aligned with those reported by Houri et al., who analysed six studies involving 1099 paediatric patients with suspected pneumonia and reported a pooled sensitivity of 90.9% (95% CI: 85.5–94.4%) and specificity of 80.7% (95% CI: 63.6–91.0%). In comparison, our analysis, which included 30 studies and a more diverse patient population, yielded a comparable pooled sensitivity of 91% (95% CI: 87–94%) but a higher specificity of 90% (95% CI: 83–94%), with low heterogeneity. This difference may be attributable to methodological factors. Houri et al. reported inconsistent operator training, potentially introducing performance bias, and their specificity estimates showed high variability. By contrast, our study applied inclusion criteria regarding operator expertise and setting, thereby achieving greater diagnostic consistency.

When compared with other meta-analyses [1,19,64,65,66,67], our study demonstrated much lower heterogeneity (I^2^ = 8.5% vs. 52–85% in previous reviews). This consistency likely reflects the exclusion of studies focused on neonatal population, the emphasis on standardised comparators (mostly CXR or CT), and the inclusion of studies from healthcare systems with more uniform protocols. However, some variability in Se and Sp persisted across studies. While the pooled heterogeneity statistic was low, this primarily reflects statistical consistency and may not capture remaining methodological differences. Although most studies used a linear probe and comprehensive multi-zone scanning, variability persisted in exact scanning protocols, image interpretation criteria, and operator experience—factors that could influence diagnostic performance in practice.

Studies with smaller or more diverse populations exhibited wider intervals. Despite this variability, the pooled estimates confirmed LUS as a reliable diagnostic tool across clinical settings, with Se values clustering towards the higher end, indicating consistent pneumonia detection. CXR showed greater variability, suggesting that LUS provides more stable diagnostic accuracy than traditional imaging methods.

Operator expertise remains a key factor influencing LUS accuracy. Experienced clinicians consistently report higher Se and Sp compared to novices, as demonstrated in studies as Tsou et al. [65] and Guitart et al. [17]. Inexperienced operators, by contrast, tend to underestimate findings, leading to reduced sensitivity [48]. Interobserver agreement also decreases with less training, highlighting the need for standardised education and competency assessment [68]. The implementation of structured training programmes, as suggested by Orso et al., would improve diagnostic consistency and ensure reliable performance across different clinical settings [16].

Beyond initial diagnosis, LUS provides added value by facilitating longitudinal monitoring. Several studies have shown its feasibility in tracking consolidations and pleural effusions, guiding antibiotic stewardship, and supporting timely clinical decisions without exposing children to repeated radiation [46,53]. This feature is particularly important in paediatric intensive care units, where patients often require frequent reassessments and radiation-free imaging becomes more sensitive.

Overall, this meta-analysis reinforces LUS as a safe, accurate, and versatile tool for diagnosing paediatric pneumonia. Its portability, non-invasive nature, and strong diagnostic performance make it especially valuable in emergency and critical care environments, as well as in resource-limited settings where access to advanced imaging may be restricted. By enabling earlier and more precise diagnosis, LUS can improve clinical outcomes, reduce unnecessary antibiotic use, and optimise resource utilisation.

## 5. Limitations

This meta-analysis has limitations that should be considered when interpreting the findings. Although heterogeneity was lower than in previous reviews, some variability persisted across included studies. This likely reflects differences in study design, patient demographics, operator expertise, and ultrasound protocols. The lack of standardised criteria for LUS interpretation across studies may have further contributed to variability. Operator dependence remains a key issue, while experienced clinicians demonstrated consistently high diagnostic accuracy, results were less reliable when LUS was performed by less trained operators. In addition, the presence of publication bias cannot be excluded, Egger’s test was statistically significant (*p* < 0.005), and visual funnel plots (Supplementary Material, Figure S1) suggest that studies reporting higher diagnostic accuracy were more likely to be published, potentially leading to a modest overestimation of LUS performance.

Finally, variability in comparator methods, such as reliance on clinical diagnosis in some studies versus CXR or CT in others, may have led to the overestimation or underestimation of accuracy. Although the bivariate model accounts for inter-study variability, the use of non-uniform reference standards (clinical, CXR, CT, or composite criteria) remains a limitation that could influence the pooled accuracy estimates.

## 6. Conclusions

This systematic review and meta-analysis provides strong evidence that LUS is a highly accurate and reliable tool for diagnosing pneumonia in critically ill paediatric patients. Future research should focus on standardising diagnostic criteria and follow-up. By addressing these aspects, LUS could be fully established as a first-line imaging modality for paediatric pneumonia, improving diagnostic certainty and patient outcomes.

## Figures and Tables

**Table 1:** Demographic characteristics.

Author	Year	Country	Study Type	Sample	Gender (Female)	Age Range	Setting	Comparator
R. Copetti [35]	2008	Italy	Cohorts	79	-	6 month–16 years	ER	CXR/CT
J. Kurian [36]	2009	United States	Cohorts	19	53%	8 month–17 years	ER	CT
D. Iuri [37]	2009	Italy	Cohorts	28	39%	0–17 years	ER	CXR
V.P. Shah [38]	2013	United States	Cohorts	200	44%	0–21 years	ER	CXR
V.A. Caiulo [39]	2013	Italy	Cohorts	102	-	1–16 years	Ward	CXR
S. Esposito [40]	2014	Italy	Other	103	46%	1 month–14 years	PICU	CXR
F. Reali [41]	2014	Italy	Cohorts	107	43%	≤16 years	Ward	CXR
T.I. Dianova [42]	2015	Russia	Cohorts	154	44%	<18 years	Ward	CXR+ CT + Clinical
E. Urbankowska [43]	2015	Poland	Cohorts	106	37%	1 month–18 years	ER	CXR
M.C. Ho [44]	2015	Taiwan	Cohorts	163	44%	Paediatric (median age 73.2 month)	Ward	CXR
G. Iorio [45]	2015	Italy	Cohorts	52	-	2 month–12.5 years	Ward	CXR + Clinical
M. Guerra [46]	2016	Italy	Cohorts	222	-	3 month–16 years	ER	CRX
S. Ianniello [47]	2016	Italy	Cohorts	84	48%	3–16 years	ER	CXR
C. Zhan [48]	2016	China	Other	82	43%	0–15 years	ER	CXR
T. Varshney [49]	2016	United States	Cross-sectional	94	-	>2 years	ER	-
A.S. Claes [50]	2017	Belgium	Other	143	46%	0–16 years	ER	CXR
H. Levent [51]	2017	Turkey	Observational prospective study	160	45%	1 month–18 years	ER	CXR
C. Boursiani [52]	2017	Greece	Cohorts	69	60%	6 month–12 years	ER	CXR
K. Kumar [53]	2017	India	Cohorts	118	45%	2–59 month	Ward	CXR
S. Saraya [54]	2017	Egypt	Other	56	52%	4 month–10 years	ER	CT
S. Claudiu [55]	2017	Romania	Case–control	81	48%	Paediatric	Ward	CXR
L.E. Ellington [56]	2017	United States	Cohorts	1300	43%	2–59 month	ER + Ward	CXR
F. Samson [57]	2018	France	Cohorts	200	42%	Paediatric	ER	CXR
M. Correa [58]	2018	Argentina	Cohorts	21	60%	<5 years	ER + Ward	-
C. Lissaman [59]	2019	United Kingdom	Cohorts	97	48%	1 month–18 years	ER	CXR
A. Kağan [60]	2019	Turkey	Observational prospective study	145	44%	<18 years	ER	Clinical + CXR
T.H. da Souza [61]	2019	Brazil	Cohorts	23	48%	<14 years	PICU + Ward	CXR
A. Cağlar [62]	2019	Turkey	Other	91	41%	0–18 years	ER	CXR
L.M. Hegazy [63]	2020	Egypt	Cross-sectional	63	52%	1 month–18 years	ER	CXR
C. Guitart [18]	2022	Spain	Intervention trial	194	58%	<18 years	PICU	CXR

**Table 2:** Description of LUS technique: equipment, operators, and results.

Author	Nº LUS	Nº Comparator	Operator	LUS Equipment and Prove	Scanning Zones	LUS Pattern	Blinding
R. Copetti [35]	79	79	Expert	Megas CVX, Esaote Medical Systems, Genova, Italy. 3.5–5 MHz convex probe and a high-resolution 7.5–10 MHz linear probe	Ant, post, lat, sup, inf	C, PE	Yes
J. Kurian [36]	19	19	Two experienced ultrasound technologists performed US, radiologist reviewed US	U22, HDI 5000 (Philips Healthcare) (Eindhoven, The Netherlands), Acuson Sequoia 512 (Siemens Healthcare) (Erlangen, Germany) Linear (5–12 MHz), convex (2–5, 4–9, or 5–8 MHz), and vector (5–8 MHz) probe	Ant, post, lat	C, PE	Yes
D. Iuri [37]	32	32	Experienced physician	ATL (Hong Kong, China), HDI 5000 units, convex probe 2–5 MHz high-resolution linear probe 5–12 MHz	Ant, post, lat, sup, inf	C, AB, PE	Yes
V.P. Shah [38]	200	200	15 paediatric emergency physicians with different degrees of expertise	Micromaxx Sonosite (Bothell, WA, USA) and GS60 Siemens (Erlangen, Germany), linear probe (7.5–10 MHz)	-	C, AB	Yes
V.A. Caiulo [39]	102	102	Experienced physician/expert	Kontron Agile, Toshiba (Kanagawa, Japan), Nemio High-resolution linear probe (6 to 12 MHz)	Ant, post, lat, sup, inf	C, AB/FB, PE	Yes
S. Esposito [40]	103	103	3 h training course	MyLab™ 25 Gold (Esaote, Genova, Italy) with a convex 2.5–6.6 MHz probe and a linear 7.5–12 MHz probe	Ant, post, lat, sup, inf	C, AB	Yes
F. Reali [41]	107	107	Expert	Mylab 25; (Esaote, Genova, Italy). Linear probe (7.5–10 MHz)	Ant, post, lat, sup, inf	C	Yes
T.I. Dianova [42]	154	154	Unspecified	Hitachi Vision Avius (Tokio, Japan) and Sonoscape s8Exp (Shenzhen, China) 4–11 mHz multifrequency linear probes and 4–11 mHz convex probes	Ant, post, lat, sup, inf	C, AB and/or pleural abnormalities	No
E. Urbankowska [43]	106	106	Paediatric sonographer	ProSound a6 ALOKA, (Tokio, Japan). Linear (5–9 MHz) and convex (3–7 MHz) probe	Ant, post, lat, sup, inf	C	Yes
M.C. Ho [44]	163	163	Expert Pulmonologist	Philips (Eindhoven, The Netherlands), (Sono 57500) Bothell, WA, USA 5 MHz convex probe	Ant, post, lat, sup, inf	C, AB/FB, PE	Yes
G. Iorio [45]	52	52	Expert	Sonosite MicroMaax Systems Bothell, WA, USA 5–10 MHz linear probe	Ant, post, lat, sup, inf	C, AB/FB, PE	Yes
M. Guerra [46]	222	222	Expert	MyLAB 25, Esaote Medical Systems, (Genova, Italy). A high-resolution (7.5–10 MHz) linear probe and 3.5–5 MHz convex probe	Ant, post, lat, sup, inf	C, AB	Yes
S. Ianniello [47]	84	84	Unspecified	Siemens Acuson Seuoia 512 system (Erlangen, Germany). Curved array 4 Mhz multifrequency probe and lineal probe (7.5–10 Mhz)	Ant, post, lat, sup, inf	C, AB/FB, PE	No
C. Zhan [48]	164	164	3 h training course	Sonosite Titan scanner (Bothell, WA, USA) with a 5–10 MHz linear array transducer, and GE LOGIQe with a 5- to 13- MHz	Ant, post, lat, sup, inf	C, AB, PE	Yes
T. Varshney [49]	94	0	2 operators (one novice and one senior)	Zonare, (Mountain View, CA, USA). linear ultrasound probe (L14–3 MHz)	Ant, post, lat, sup, inf	C	Yes
A.S. Claes [50]	143	143	Senior	Philips iU-22. (Eindhoven, The Netherlands) Linear probe (L 12–5 MHz) and medium frequency convex probe (C 9–4 MHz)	Ant, post, lat, sup, inf	C	No
H. Levent [51]	160	160	Expert	SonoSite (Bothell, WA, USA) Edge ultrasound device with 6–13 MHz linear probe	Ant, post, lat, sup, inf	C, AB/FB, PE	Yes
C. Boursiani [52]	69	69	Expert (>25 years of experience)	5–8 MHz micro convex, 5–12 MHz linear array, and 3–5 MHz convex transducers	Ant, post, lat, sup, inf	C, PE	Yes
K. Kumar [53]	118	118	No expert	LOGIQ P5 ultrasound(GE, Boston, MA, USA), High-resolution micro-convex transducer with the depth of 8 cm	Ant, post, lat, sup, inf	C, AB, FB, PE	No
S. Saraya [54]	56	56	Radiologist	LOGIC S8 (GE, Boston, MA, USA) health- care ultrasound Linear (6–12 MHz) ± convex (2–5 MHz) probe	Ant, post, lat, sup, inf	C, AB	Yes
S. Claudiu [55]	81	81	Senior radiologist	Accuviz V20 Medison US (Samsung, Seoul, Republic of Korea) since 2014, after that Toshiba Xario 200 US Convex probe (7–11 MHz) and linear probe (3,5–5 MHz)	Ant, post, lat, sup, inf	C, AB, PE	No
L.E. Ellington [56]	1062	1138	Paediatrician trained in LUS/Paediatric radiologist	Micromaxx portable ultrasound medicine (Sonosite, Bothell, WA, USA) HFL38/13–6 MHz linear transducer	Ant, post, lat, sup, inf	C, PE	Yes
F. Samson [57]	200	200	Paediatrician, 3 h training course	S-Nerve Sonosite (Sonosite, Bothell, WA, USA) 6–15 MHz linear probe	Ant, lat	C, AB	Yes
M. Correa [58]	21	15	Vectors/brightness	Ultrasonix SonixTouch (GE, Boston, MA, USA) Linear prove L14/38	-	Artificial intelligence methods	Yes
C. Lissaman [59]	97	97	Physician/ Paediatrician	Zonar Z.one ultra L 14–5 MHz linear transducer	Ant, post, lat, sup, inf	C, AB	Yes
A. Kağan [60]	145	120	Expert (>200 LUS)	Mindray M5, Mindray Nanshan, Shenzhen, China. 5–10 MHz linear and 2.5–5 MHz curved probes	Ant, post, lat, sup, inf	C, AB/FB, PE	Yes
T.H. da Souza [61]	23	23	5 years focus experience	GE Healthcare Vivid Q (GE, Boston, MA, USA) 5–13 MHz linear probe	Ant, post, lat, sup, inf	C, PE	Yes
A. Cağlar [62]	91	91	Unspecified	Philips ClearVue 350 (Philips, Eindhoven, The Netherlands) Linear (L12–4 MHz) and curved (C5- 1 MHz) probes	Ant, post, lat, sup, inf	C, AB	No
L.M. Hegazy [63]	63	63	Unspecified	-	Ant, post, lat, sup, inf	C	Yes
C. Guitart [18]	96	98	By intensive care physicians with special training in LUS and had at least 3 years of experience	A 12-Mhz linear probe	Ant, post, lat, sup, inf	C, AB	Yes

AB: Air bronchogram. C: Consolidation. FB: Fluid bronchogram. PE: Pleural effusion.

**Table 3:** Diagnostic accuracy of LUS for pneumonia.

	Patients	Pneumonia	True Positive	False Positive	False Negative	True Negative
R. Copetti [35]	79	60	60	0	0	19
J. Kurian [36]	19	19	18	0	1	0
D. Iuri [37]	28	24	22	0	2	4
V.P. Shah [38]	200	64	55	17	9	129
V.A. Caiulo [39]	102	89	88	0	1	13
S. Esposito [40]	103	48	47	3	1	52
F. Reali [41]	107	81	76	1	5	25
T.I. Dianova [42]	154	154	147	0	7	0
E. Urbankowska [43]	106	76	71	0	5	30
M.C. Ho [44]	163	163	159	0	4	0
G. Iorio [45]	52	29	28	1	1	22
M. Guerra [46]	222	214	207	0	7	8
S. Ianniello [47]	84	61	60	0	1	23
C. Zhan [48]	82	82	33	7	49	75
T. Varshney [49]	94	8	8	33	0	53
A.S. Claes [50]	143	45	44	8	1	90
H. Levent [51]	160	149	142	4	7	7
C. Boursiani [52]	69	66	62	0	4	3
K. Kumar [53]	118	118	105	0	13	0
S. Saraya [54]	56	36	26	1	10	19
S. Claudiu [55]	81	72	57	5	15	4
L. E. Ellington [56]	1300	191	169	0	22	230
F. Samson [57]	200	85	74	6	11	109
M. Correa [58]	21	15	14	0	1	6
C. Lissaman [59]	97	44	40	17	4	36
A. Kağan [60]	145	43	35	0	8	102
T.H. da Souza [61]	23	21	20	2	1	0
A. Cağlar [62]	91	56	55	15	1	20
L.M. Hegazy [63]	63	30	28	1	2	32
C. Guitart [18]	194	97	76	2	21	95

**Table 4:** Pooled estimates at a 95% confidence interval, including sensitivity, specificity, positive and negative likelihood ratio (LR), and the log-diagnostic odds ratio (lnDOR). Heterogeneity assessed using Higgins’ I^2^ statistic. LUS: lung ultrasound.

LUS				Comparator		
	Estimate	Conf. Low	Conf. High	Estimate	Conf. Low	Conf. High
Sensitivity	0.9085	0.8664	0.9383	0.8813	0.8466	0.9089
Specificity	0.9001	0.8343	0.9416	0.9086	0.833	0.9519
posLR	9.44	5.48	15.5	10.2	5.28	18.2
negLR	0.104	0.0688	0.149	0.132	0.101	0.17
lnDOR	4.557	3.795	5.193	4.368	3.578	5.024
I2	0.08475	0.04977	0.08671	0.03539	0.0252	0.03583

## Data Availability

The raw data supporting the conclusions of this article will be made available by the authors on request.

## Supplementary Materials

The following supporting information can be downloaded at: https://www.mdpi.com/article/10.3390/diagnostics15243122/s1, Figure S1. Funnel plots; Figure S2. QUADAS-2 Risk of Bias Assessment.
