# Supplementary material for: Diagnostic Accuracy of Lung Ultrasound for Pneumonia in Acutely and Critically Ill Neonates, Children, and Young Adults: A Systematic Review and Meta-Analysis
Source: Diagnostics (Basel). 2025 Dec 8;15(24):3122. doi: 10.3390/diagnostics15243122 (PMC12732246; doi:10.3390/diagnostics15243122)

Supplementary material

Figure S1. Funnel plots

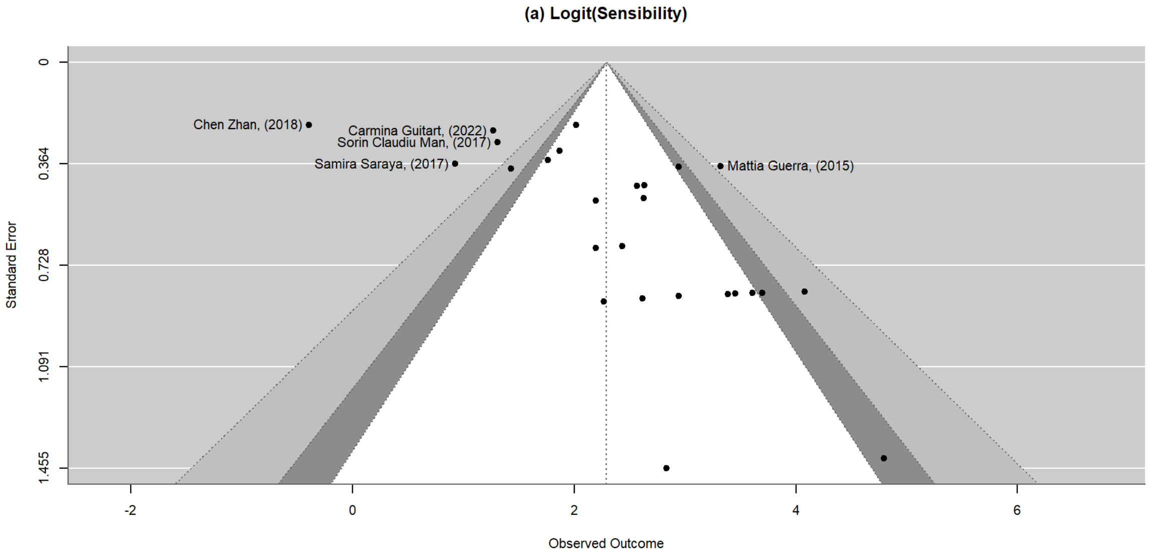

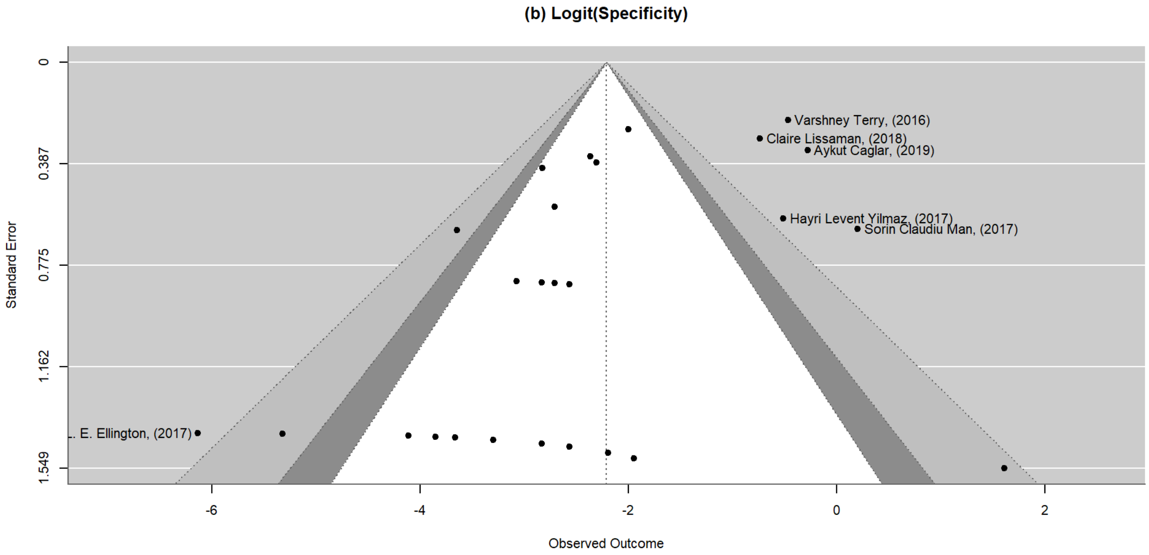

Figure S2. QUADAS-2 Risk of Bias Assessment

QUADAS-2 Risk of Bias Summary

Domain	N = 30[1]
Patient Selection	
High Risk	7 (23%)
Low Risk	21 (70%)
Unclear Risk	2 (6.7%)
Index Test	
High Risk	1 (3.3%)
Low Risk	24 (80%)
Unclear Risk	5 (17%)
Reference Standard	
High Risk	1 (3.3%)
Low Risk	23 (77%)
Unclear Risk	6 (20%)
Flow and Timing	
High Risk	1 (3.3%)
Low Risk	22 (73%)
Unclear Risk	7 (23%)

[1] n (%)

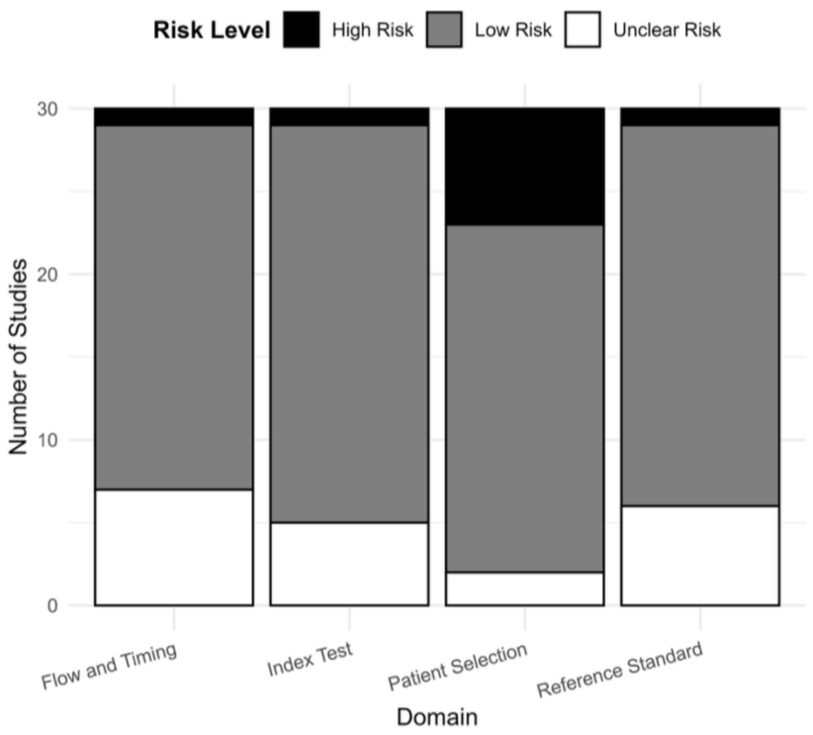

Supplement: Supplementary file 1 [file diagnostics-15-03122-s001.zip › diagnostics-3924277-supplementary.pdf]
